# Attitudes, Sentiments, and Concerns About Inclusive Education of Teachers and Teaching Students in Spain

**DOI:** 10.3389/fpsyg.2020.00521

**Published:** 2020-04-07

**Authors:** Diego Navarro-Mateu, Jacqueline Franco-Ochoa, Selene Valero-Moreno, Vicente Prado-Gascó

**Affiliations:** ^1^Department of Educational Psychology and Special Educational Needs, Faculty of Psychology, Teaching and Educational Sciences at the Catholic University of Valencia, Valencia, Spain; ^2^Department of Personality, Assessment and Psychological Treatments, Faculty of Psychology, University of Valencia, Valencia, Spain; ^3^Department of Social Psychology, Faculty of Psychology, University of Valencia, Valencia, Spain

**Keywords:** inclusive education, university students, teachers, SACIE-R, social dominance, empathy

## Abstract

The Sentiments, Attitudes, and Concerns About Inclusive Education Revised scale was developed to close the existing gap in measuring perceptions of inclusive education in the educative context. It has been widely used in other cultures but not in Spain. Our objective has been to analyze the psychometric properties in the Spanish sample by studying their relationship with empathy and social dominance variables, finally taking into account sociodemographic variables to observe if there are differences. The scale was applied to a total of 647 subjects: 323 university-students (18–45 years) and 324 in-service teachers (35–58 years). The scale showed psychometric properties suitable for the general group, the students, and the teachers. Likewise, the female students showed a more positive attitude toward inclusion, and these attitudes were associated with empathy and social dominance. This version of the Sentiments, Attitudes, and Concerns about Inclusive Education Revised is a useful tool for measuring the inclusive attitudes of undergraduate education students and in-service teachers.

## Introduction

### Inclusive Education

The inclusive approach refers to the type of education that allows students with diverse needs to find their own place and to receive their education in regular schools and classrooms (Ebersold and Watkins, 2011). Countries from all around the world have decided that, by 2030, inclusive and quality education will be guaranteed for all, thus reducing the negative impact on the emotional well-being of the students (Tardi, 2012).

Traditionally, persons with disabilities (Groce et al., 2011; Schneider et al., 2016) have been subject to discrimination, school segregation (Kim et al., 2016), as well as deep-seated forms of exclusion (Tenenbaum et al., 2017). Among the main consequences of the phenomenon of exclusion is the erosion of the well-being and health of the adults and children who suffer from it (UNICEF, 2013), profoundly affecting the quality of life of individuals as well as the equity and cohesion of society as a whole (Bodvin et al., 2018). Currently, however, this paradigm is changing: there is a shift toward understanding diversity from a social perspective (Díaz Velázquez, 2010) of human rights or inclusion as leading to respectful responses toward people both with and without disabilities (Tardi, 2012).

### Attitudes Toward Inclusive Education

As the theory of planned behavior affirms (Ajzen, 2005), the attitudes of individuals constitute the best predictor of their behavior. In this sense, one of the aspects that most frequently seems to positively affect inclusive education (IE) in the classroom is related to the attitudes and beliefs of teachers, whether they are professionally in-service teachers (de Boer et al., 2011; Chen, 2018; Glock et al., 2018) or future teachers (Sharma et al., 2014). In addition, attitudes are not innate but learned (Ajzen, 2005). Therefore, they should be taught to teachers because they, together with families, have the responsibility of educating and training children (Schneider and Coleman, 2018).

In this regard, the attitude of the different education professionals is a key factor in the development of inclusion (de Boer et al., 2011; Vaz et al., 2015; Gutentag et al., 2018). Attitudes toward inclusion have been studied in in-service teachers (Boyle et al., 2013; Collins, 2013) and in future teachers (teaching students) (Beacham and Rouse, 2012; Kuyini et al., 2018; Saéz-Gallego et al., 2019). In order to achieve inclusion, it is not enough that teachers (Messiou, 2017) or future teachers (Loreman et al., 2014; Sharma and Sokal, 2015) have good knowledge of the educational needs of students. They must also have certain attitudes, abilities, skills and strategies that, applied in their educational praxis, enable quality education for all (Loreman et al., 2014; Novo-Corti et al., 2015). Therefore, as Gale et al. (2017) argue, it is important to understand and monitor teachers’ attitudes toward diversity (Loreman et al., 2014). Thus, the type of response, positive or negative, that the teacher (Booth and Ainscow, 2015) or future teacher (Novo-Corti et al., 2015) has toward the object of attitude—in this case diversity—will depend on the combination of the three integrating components of the attitude: cognitive (positive or negative beliefs toward diversity), affective (evaluation and positive or negative valuation toward diversity), which translates into an acceptance (inclusion) or rejection (exclusion), and conative-behavioral responses (disposition toward diversity depending on the other two components) (Ajzen, 2005; Fishbein and Ajzen, 2011).

### Factors Influencing Attitudes Toward Inclusive Education

Thus, various investigations have attempted to analyze the attitudes of teachers (de Boer et al., 2011; Booth and Ainscow, 2015) and future teachers in the educational field (Novo-Corti et al., 2015). The studies explain that factors, such as gender, age, experience, training on attention to people with disabilities (Forlin and Chambers, 2011; Loreman et al., 2014; Schmidt and Vrhovnik, 2015) as well as types and levels of disability in children (Forlin and Chambers, 2011), could be influenced in the IE.

In the case of teachers, studies suggest that both work experience and contact positively affect teachers’ inclusive attitudes (Tsakiridou and Polyzopoulou, 2014), while some older and more experienced teachers appear to tend toward the opposite (Loreman et al., 2007). On the other hand, university students’ contact with people with disabilities is crucial for inclusion. In this sense, it improves and modifies beliefs and attitudes toward them (Mellado et al., 2017) at the same time as it enables the students to form comprehensive conceptions of them (Novo-Corti et al., 2015). Regarding gender, its influence on these types of attitudes seems to be controversial. While studies, such as those by Efilti and Arslan (2017), found no significant effect of gender on teachers’ attitudes toward inclusion, some studies have indicated that women have more positive attitudes toward educational inclusion (Tsakiridou and Polyzopoulou, 2014; Shatri, 2017), whereas other studies (de Boer et al., 2011) found more positive attitudes among male teachers. All this controversy may be due to the very measurement instruments used to measure attitudes toward inclusion—instruments that have rarely been adapted and validated for the specific context in which they are being used. In the case of future students, the influence of these factors seems clearer, with studies suggesting that women tend to have more positive attitudes toward students with disabilities than men (Novo-Corti et al., 2015). It seems that beliefs and attitudes toward diversity can facilitate or hinder inclusion in the educational context (Booth and Ainscow, 2015; Messiou et al., 2016); therefore, their adequate measurement is highly relevant.

Moreover, there are other socials factors (Irby and Clough, 2015), such as the culture (Armstrong et al., 2016), policy, and practices in schools; i.e., the implementation of inclusion in schools, resources and their distribution, the support of school administration and teachers themselves, and the organizational framework (teacher role or supports available in the classroom) (Galović et al., 2014; Schmidt and Vrhovnik, 2015). In addition, such attitudes may also be affected by the contact that individuals have had with people with special needs (Crowson and Brandes, 2014; Gonzalez et al., 2015). However, few studies have analyzed the differences in attitudes toward the inclusion of teachers and teacher training students as a function of these type of variables.

On the other hand, one of the variables that can influence the development of IE is whether one is a teacher training student or an in-service teacher. Some studies about future teachers (teacher training students) affirm that they feel that they do not receive adequate training on educational inclusion (Tsakiridou and Polyzopoulou, 2014), which leads to a decrease in confidence in the design of inclusive curricular activities and programs (de Boer et al., 2011), although it seems that students do have a positive attitude toward the concept of inclusion (Lambe et al., 2013). Other studies have found that younger teachers tend to adopt more favorable views toward the inclusion of students with special educational needs (SENs) (Forlin et al., 2011). However, we have not been able to observe studies that simultaneously analyze such inclusive attitudes in both groups in the same study, and we therefore cannot determine which group has more positive attitudes toward inclusion: in-service teachers or students.

### Tools on Inclusive Education

During the last few years, there has been a proliferation of tools to measure attitudes toward the inclusion of students with educational needs in the Spanish context (Loreman et al., 2014; Abellán et al., 2017). Some examples of these include the Teacher Education for Inclusion Assessment Questionnaire (CEFI) (González-Gil et al., 2013), the Questionnaire on Attitudes Toward IE for Teachers in Training (Vélez, 2013), or the most recent Questionnaire for Future Teachers of Secondary Education on the Perceptions About Attention to Diversity (Ruiz and Palomino, 2015). Although these scales have been used in the Spanish context, they do not focus on specifically measuring attitudes, feelings, and concerns toward inclusive education; they also focus on other aspects, such as training or inclusion needs. Internationally, the probably most common scale in use is the Sentiments, Attitudes, and Concerns about Inclusive Education (SACIE) scale, created in 2007 (Loreman et al., 2007), which, revised 4 years later, was subsequently named SACIE-R (Forlin et al., 2011).

This scale consists of 15 items grouped into three factors. The first factor (Sentiments) evaluates the sentiments in the interaction or contact with students with disabilities or with students cataloged by the educational system with SENs; the second factor (Attitudes) focuses on the acceptance of these students; and the last factor (Concerns) evaluates concerns about IE education (Loreman et al., 2007). The scale has been used with working teachers and teachers in training (Loreman et al., 2007; Forlin et al., 2011; Cansiz and Cansiz, 2018; Murdaca et al., 2018), although neither of groups have been compared in the same study before.

Since its inception, SACIE-R has been adapted and validated to different cultural contexts, such as Italian (Murdaca et al., 2018), Turkish (Cansiz and Cansiz, 2018), and Portuguese (Santos and César, 2010). However, so far, it has never been adapted and validated for the Spanish context. Likewise, the existing studies have been carried out mostly with teachers, and the scale has thus never been analyzed simultaneously in both in-service teachers and future teachers (teachers in training).

Therefore, the objective of this study was twofold: on the one hand, the aim was first to adapt and validate the SACIE-R scale to Spanish in a sample of university students and teachers; on the other hand, the aim was to observe the attitudes toward inclusion of in-service teachers and teacher training students in Spain while at the same time analyzing the effects of gender, training, or the level of contact have those attitudes.

## Materials and Methods

### Participants

The participants were recruited using convenience sampling methods. The inclusion criteria for the case of the teachers were to be active at the time of the data collection, to exercise the profession at non-university levels, to not have any type of disability or illness preventing them from completing the questionnaire on their own, and to accept participation in the study. In the case of the students, the criteria were belonging to the last course of the degree in teaching or being a post-graduate student, not working as teachers at the time of the data collection, not having any type of disability or illness that would prevent them from completing the questionnaire on their own, and accepting participation in the study. The sample consisted of 647 subjects where 323 were university students studying teaching (49.92%). The students were in the last year of their degree, and most of them were studying for a degree in Primary Education (52.56%), followed by those studying for a double degree in Pre-school and Primary Education (31.73%) and those studying for a degree in Pre-school Education (14.74%). All of the students belonged to a medium socioeconomic level. The other 324 (50.08%) were teachers of the various educational stages (non-university levels): pre-elementary, primary, and secondary education. According to the educational stages in which they teach: 29% teach in pre-school education, 73.1% in primary education, 19.4% in secondary education, and 7.1% in high school education. The average age of the group of respondents was 32.42 years (*SD* = 12.32), with a minimum age of 18 and a maximum of 64 years. The percentage of women was 71.60%. The students’ subsample (323), consisted of individuals aged between 18 and 45 years (*M* = 21.20, *SD* = 6.37), with 77.2% being women (*n* = 249). In comparison, ages in the active teacher subsample (324) ranged between 35 and 58 years (*M* = 41.79, *SD* = 10.10), and the percentage of women was 66% (*n* = 214). The teachers’ teaching experience ranged from 0 to 39 years (*M* = 19.72, *SD* = 90.12). The number of teachers that claimed to have received training on diversity attention quite or very often during their career was 30.09%, while 20.38% said they had occasionally received this type of training, and 49.53% stated that they had never or rarely received it. Regarding the students, nearly half of those interviewed (47.95%) stated they had received training on diversity quite or very often during their studies, while 30.48% had occasionally received this type of training, and 21.56% said that they had never or rarely received it.

In terms of the frequency with which teachers had had their diversity training updated, 36.99% said they had often or very often been updated, 36.36% stated they had occasionally been updated, while 26.65% claimed they had never or rarely been updated. As for the students, 36% had often or very often been updated, 30% had occasionally been updated, while 34% had never or rarely been updated.

Regarding the frequency with which teachers had taught students with disabilities, 40.44% had often or very frequently taught these types of students, 32.29% indicated that they had occasionally taught them, and 27.27% indicated that they had never or rarely taught students with disabilities. In the case of students, 20.09% had often or very frequently taught this type of pupils, and the same percentage indicated that they had occasionally taught them, while 59.82% stated that they had never or rarely taught pupils with disabilities.

Finally, about half of the students (50.32%) claimed to have had peers with SENs during their schooling, while 49.68% reported not having had peers with SENs. Furthermore, 48.25% indicated that they had a family member or acquaintance with some type of disability, while 51.75% stated that they did not.

### Measures

SACIE-R: adaptation of the SACIE-R (Forlin et al., 2011) scale to the Spanish context was carried out by the research team. The scale assesses three dimensions of IE: Sentiments, Attitudes, and Concerns. This questionnaire consists of 24 items to be answered with a five-level Likert item (1 = strongly disagree, 2 = disagree, 3 = neither agree nor disagree, 4 = agree, and 5 = strongly agree). The scale showed an acceptable internal consistency (α > 0.74) within previous studies (Forlin et al., 2011).

An *ad hoc* questionnaire was carried out to collect the variables of gender, age, specific training in the face of diversity, frequency of updating educational training, any experience or contact with students with diversity, and the presence of a relationship with peers or family members with diversity.

### Procedure

The SACIE-R scale was adapted according to the guidelines of the International Testing Commission (ITC) (Muñiz et al., 2013). For the language contextual adaptation of the instrument, two native linguists translated and back-translated (English–Spanish–English) the items of the scale; this process was complemented with the review of the resulting items by experts in IE, which ensured the adequacy of the statements for the Spanish context (Table 1). Next, the participants were selected: both the university students and the teachers of the different educational stages from the Valencian Community. The collection of both samples was carried out over 3 months. In the case of the student sample, the information was collected during school hours and during the time allowed by the university professors (approximately 20 min) who voluntarily facilitated the interruption of the class session in order to explain and proceed to collect the sample voluntarily from the student teachers who placed their questionnaire in a blank envelope. This sample was of convenience to students of teaching at the Catholic University of Valencia. The student response rate was 100%. In the case of the sample of teachers, 10 centers that voluntarily wanted to participate were provided with the total number of questionnaires requested and some hermetically sealed boxes in which the questionnaire could be deposited, thus guaranteeing anonymity among the participating subjects. The response rate from teachers was 45%. Due to the nature of the study, participants answered voluntarily, and the questionnaires were anonymous. The study was approved by the bioethics committee of the Catholic University of Valencia (PRUCV/2015/660). All participants received detailed information about the aims and procedures and were informed of confidentiality.

**TABLE 1:** Scale of sentiments, attitudes, and concerns toward inclusive education (Spanish version adapted from the SACIE-R scale).

(1)Totalmente en desacuerdo	(2) En desacuerdo	(3) Ni de acuerdo ni en desacuerdo	(4) De acuerdo	(5) Totalmente de acuerdo
(1) Me da miedo mirar directamente a la cara a una persona con discapacidad.				
(2) Los alumnos que tienen dificultad para expresar verbalmente sus pensamientos deberían estar en aulas ordinarias.				
(3) Los alumnos que necesitan una Adaptación Curricular Individualizada Significativa (ACIS) deberían estar en aulas ordinarias.				
(4) Los alumnos que necesitan Sistemas Aumentativos y Alternativos de Comunicación (SAAC)				
(Por ejemplo, Braille o lenguaje de signos) deberían estar en un aula ordinaria.				
(5) Los alumnos que tienen dificultades para prestar atención deberían estar en aulas ordinarias.				
(6) Los alumnos que suspenden frecuentemente los exámenes deberían estar en aulas ordinarias.				
(7) Me preocupa que, si tengo alumnos con discapacidad en mi clase, mi trabajo se incrementará.				
(8) Me preocupa no tener los conocimientos y habilidades suficientes para atender a los alumnos con discapacidad.				
(9) Me preocupa lo difícil que será dar una atención apropiada a todos los alumnos en una clase inclusiva.				
(10) Me preocupa que los alumnos con discapacidad no sean aceptados por el resto de la clase.				
(11) Me preocupa que, por tener alumnos con discapacidad en mi clase, yo estaré más estresado/a.				
(12) No quiero ni pensar que algún día yo pueda acabar teniendo una discapacidad.				
(13) Intento que los contactos con personas discapacitadas sean cortos y busco acabarlos lo antes posible.				
(14) Me sentiría fatal si tuviera una discapacidad.				
(15) Me es difícil superar el impacto que siento al conocer a personas con discapacidad física severa.				

### Statistical Analysis

The statistical analysis was carried out with the programs SPSS Statistics (version 24), EQS (version 6.3) (Satorra and Bentler, 2001; Crawford and Henry, 2003) and FACTOR (Lorenzo-Seva and Ferrando, 2006). First, the descriptive statistics for each item were calculated. Second, the reliability and validity of the scale for the whole sample and for each subsample was analyzed. Third, Pearson correlations were performed to determine the relationship between the different dimensions of the questionnaire. The instrument’s invariance was calculated, and then descriptives comparing both samples were calculated. Finally, the influence of gender, training, and contact played on the attitude toward inclusion was evaluated by means of *t*-test and Pearson correlations.

## Results

### Adaptation and Validation of the SACIE-R Scale

In order to examine the reliability of the scale, its internal consistency was calculated using Cronbach’s alpha. However, since this index does not consider the influence on the other reliability construction, both the composite reliability coefficient (CRC) and the average variance extracted (AVE) (Fornell and Larcker, 1981) were calculated. The minimum CRC value was 0.70, which was considered adequate, as (Nunally, 1978) values higher than 0.50 are recommended for the AVE (Bagozzi and Yi, 1988), although others studies have considered that values of AVE higher than 0.40 are adequate too (Vila et al., 2000).

The questionnaire showed acceptable scores for overall reliability for the whole sample and for the different subsamples (in-service teachers and university students). The values of the different dimensions of the SACIE-R ranged between α = 0.64–0.83, CRC = 0.63–0.84 and AVE = 0.37–0.52 for the different dimensions, fairly acceptable values as indicated by the literature (Fornell and Larcker, 1981; Vila et al., 2000). The analysis of internal consistency suggested the elimination of item 1 in the Sentiments dimension and item 10 in the concerns dimension because the factorials loads were below 0.40 (Table 2).

**TABLE 2:** Reliability analysis for the total set and for the subsamples separately.

Ítems	*M*			*SD*			r_jx_			α-x			S			K		
	W	S	T	W	S	T	W	S	T	W	S	T	W	S	T	W	S	T
**Attitudes: Wα = 0.83; Sα = 0.82; Tα = 0.83; CRC_W_ = 0.83; AVE_W_ = 0.51; CRC_S_ = 0.83; AVE_S_ = 0.50; CRC_T_ = 0.84; AVE_T_ = 0.52**																		
I2	3.84	3.72	3.96	1.13	1.25	0.98	0.49	0.48	0.50	0.83	0.83	0.84	–0.79	–0.69	–0.76	0.01	–0.47	0.38
I3	4.03	3.86	4.19	1.04	1.15	0.89	0.63	0.60	0.68	0.79	0.79	0.79	–1.00	–0.77	–1.17	0.42	–0.34	1.53
I4	3.82	3.83	3.82	1.10	1.16	1.03	0.74	0.70	0.62	0.76	0.76	0.81	–0.73	–0.82	–0.59	–0.14	–0.16	–0.20
I5	4.15	4.11	4.19	1.00	1.12	0.86	0.61	0.72	0.77	0.80	0.75	0.77	–1.25	–1.28	–1.00	1.22	0.86	0.89
I6	4.22	4.17	4.28	1.03	1.09	0.95	0.66	0.59	0.64	0.78	0.79	0.80	–1.37	–1.36	–1.32	1.29	1.13	1.25
**Sentiments: Wα = 0.64; Sα = 0.64; Tα = 0.65; CRC_W_ = 0.69; AVE_W_ = 0.45; CRC_S_ = 0.72; AVE_S_ = 0.47; CRC_T_ = 0.67; AVE_T_ = 0.43**																		
I1	1.59	1.71	1.47	1.01	1.11	0.89	0.21	0.18	0.31	0.66	0.69	0.63	1.73	1.51	1.96	2.08	1.25	3.07
I12	3.22	3.10	3.34	1.38	1.49	1.26	0.50	0.50	0.49	0.52	0.55	0.54	–0.27	–0.12	–0.40	–1.12	–1.37	–0.76
I13	1.46	1.37	1.55	0.79	0.78	0.79	0.36	0.39	0.32	0.61	0.62	0.62	1.72	2.26	1.28	2.36	4.72	0.73
I14	3.04	2.70	3.37	1.29	1.31	1.18	0.49	0.54	0.45	0.53	0.52	0.56	–0.10	0.21	–0.34	–1.01	–1.01	–0.71
I15	2.17	2.25	2.09	1.09	1.15	1.04	0.43	0.46	0.42	0.57	0.57	0.58	0.65	0.53	0.76	–0.44	–0.71	–0.09
**Concerns: Wα = 0.64; Sα = 0.64; Tα = 0.68; CRC_W_ = 0.67; AVE_W_ = 0.42; CRC_S_ = 0.68; AVE_S_ = 0.43; CRC_T_ = 0.63; AVE_T_ = 0.37**																		
I7	2.26	1.98	2.54	1.24	1.09	1.32	0.40	0.33	0.45	0.59	0.61	0.62	0.68	0.96	0.40	–0.63	0.10	0.27
I8	3.87	3.91	3.83	1.13	1.12	1.14	0.44	0.49	0.48	0.57	0.53	0.61	–0.81	–0.80	–0.81	–0.18	–0.25	0.27
I9	3.63	3.49	3.78	1.09	1.15	1.02	0.49	0.45	0.43	0.55	0.55	0.63	–0.58	–0.52	–0.59	–0.27	–0.47	0.27
I10	3.90	4.13	3.68	1.09	1.01	1.13	0.24	0.26	0.32	0.66	0.64	0.67	–0.97	–1.2	–0.78	0.39	1.15	0.27
I11	2.13	1.81	2.44	1.15	1.03	1.17	0.44	0.42	0.49	0.57	0.57	0.60	0.74	1.26	0.37	–0.42	0.91	0.27

*Sample of university students of teaching (S) and in-service teachers (T) Whole Set (W) Analyzed Items (I) reliability analysis: Mean (M), standard deviation (SD), item-total correlation (rjx), Cronbach’s alpha if the element is eliminated (α-x), asymmetry (A) and kurtosis (K); composite reliability (CRC), average variance extracted (AVE).*

The internal validity was checked by means of several exploratory factor analyses (EFA) and confirmatory factor analyses (CFA). First, the adequacy of the data for these analyses was tested using the Kaiser Meyer Olkin index (KMO) and the Bartlett sphericity test. In all cases both were adequate: for the whole sample (KMO = 0.77, χ^2^ = 2523, *df* = 91, *p* ≤ 0.001) for the students (KMO = 0.74, χ^2^ = 1324.1, *df* = 91, *p* ≤ 0.001) and, finally, for the teachers (KMO = 0.78, χ^2^ = 1418.8, *df* = 91, *p* ≤ 0.001).

Next, an EFA was performed using the unweighted least squares (ULSs) method. Likewise, the normalized direct oblimin rotation was used. However, the analysis was repeated using the normalized Varimax rotation since the correlations between the factors proposed by the EFA, with oblimin rotation, were quite low (<0.30) and did not allow for the relationship between the factors proposed by the EFA to be verified (Lloret et al., 2014).

First by performing the EFA without any restriction on the number of factors, the EFA recommended the grouping of items into four common factors. However, this solution did not offer a good theoretical interpretation so it was decided to check the adjustment of the factorial structure fixed in three dimensions (attitudes, sentiments, and concerns) derived from the proposal of the original authors of SACIE-R (Forlin et al., 2011). After the application of the EFA fixed to three factors in the whole sample, an indicator (item 1) with a saturation lower than 0.40 was eliminated. The factorial solution coincided with the whole of the sample and the subsample of students; however, in the teacher sample, some indicators saturated in a different factor and two indicators presented a factorial load lower than 0.40 (items 10 and 15). For the sample, the variance explained by the three factors was 58.55%; in the students it was 59.75% and in teachers 54.52%.

Once the EFAs had been performed, several CFAs were also carried out with the aim of, on the one hand, verifying the factorial structure extracted by the EFA (14 items grouped into three dimensions) and, on the other hand, contrasting the adjustment of the original proposal considering the 15 items grouped into three dimensions (Sentiments, Attitudes, and Concerns). The indicators of adjustment of the different proposed models are shown in Table 3. The estimation was made using Maximum likelihood (ML) estimation with Satorra-Bentler’s robust correction (Satorra and Bentler, 2001) in order to correct the absence of multivariate normality. A factorial solution with a good fit was obtained after eliminating four items from two dimensions—items eight and 10 from Concerns and items 1, 13, and 14 from Sentiments—because their factorial loads were lower than 0.40.

**TABLE 3:** Goodness-of-fit indices of the SACIE-R scale according to sample type.

Model	Sample		S-B χ^2^ (*df*)	χ^2^ (*df*)	χ^2^/*df*	RMSEA (CI)	CFI	NNFI	IFI
EFA 14 items and 3 factors	Whole sample	3 factors, 14 items	404.72 (74)	459.27 (74)	6.20	0.083 (0.075–0.091)	0.85	0.81	0.85
		3 factors, 12 items	157.48 (51)	178.14 (51)	3.49	0.057 (0.047–0.067)	0.94	0.92	0.94
	Students	3 factors, 14 items	245.22 (74)	270.87 (74)	3.66	0.085 (0.073–0.096)	0.85	0.81	0.85
		3 factors, 12 items	117.81 (51)	130.04 (51)	2.55	0.064 (0.049–0.079)	0.92	0.90	0.93
	Teachers	3 factors, 12 items	171.38 (51)	198.76 (51)	3.90	0.085 (0.071–0.099)	0.88	0.85	0.88
		3 factors, 8 items	59.34 (17)	70.75 (17)	4.16	0.088 (0.064–0.112)	0.94	0.90	0.94
CFA 15 items and 3 factors (SACIE-R proposal by Forlin et al., 2011)	Whole sample	3 factors, 15 items	479.77 (87)	536.27 (87)	6.16	0.084 (0.076–0.091)	0.82	0.79	0.82
		3 factors, 11 items	125.85 (41)	147.16 (41)	3.59	0.057 (0.045–0.068)	0.95	0.93	0.95
	Students	3 factors, 11 items	76.07 (41)	86.50 (41)	2.11	0.052 (0.033–0.069)	0.96	0.95	0.96
	Teachers	3 factors, 11 items	157.26 (41)	188.02 (41)	4.58	0.094 (0.078–0.109)	0.87	0.83	0.87
	Whole sample	3 factors, 10 items	63.25 (32)	72.90 (32)	1.98	0.05 (0.03–01.08)	0.95	0.96	0.94
	Students	3 factors 10 items	63.23 (32)	72.75 (32)	1.81	0.06 (0.04–0.08)	0.96	0.96	0.95
	Teachers	3 factors 10 items	109.71 (32)	131.27 (32)	3.42	0.08 (0.07–0.10)	0.91	0.91	0.87

*S-B = Satorra Bentler; *df* = degrees of freedom; RMSEA = Mean Quadratic Approximation Error (≤0.08); CI = RMSEA Confidence Interval; CFI = Comparative Fit Index; NNFI = Non-Normed Fit Index; IFI = Incremental Fit Index; CFI, NNFI, IFI (≥0.90); χ^2^/df (≤5.00).*

Based on the significance of the χ^2^ statistic (*p* < 0.05), an adequate model fit could not be ensured in any of the cases. However, given that this analysis is very sensitive to the sample size, other goodness-of-fit indices were applied: the χ^2^/df, showing adequate fit with values lower than 5, the Non-Normed Fit Index (NNFI), the Comparative Fit Index (CFI), the Incremental and the McDonald’s Fit Indices (IFI and MFI, respectively), values above 0.90 being indicators of good fit (Wallin and Ahlstrom, 2006), and the Root Mean-Square Error of Approximation (RMSEA) in which adequate fit scores are equal to or below 0.08. Table 3 presents a summary showing these indicators for each of the CFA.

Furthermore, in order to increase the empirical evidence for the construct validity, on the one hand, we tested the convergent validity of the scale on the basis of the results obtained in the CFA, and the items that make up the SACIE-R were significantly and strongly correlated with the latent variable they assumed to measure, oscillating from 8.49 to 12.31 (*t* ≥ 1.96) for the whole sample, from 5.70 to 9.00 for the students, and from 5.34 to 8.63 for the teachers. In all cases, the *t*-values were above 3.29 and failed to improve when new loads were included. On the other hand, discriminant validity was evaluated by means of the AVE (Fornell and Larcker, 1981; Netemeyer et al., 1990). To determine the existence of discriminant validity, we found that all correlations between the various factors were less than 0.85, and the AVE square root was higher than the correlation among the pairs of factors or dimensions considered (Fornell and Larcker, 1981; Netemeyer et al., 1990; Vila et al., 2000). The results, displayed in Table 4, suggest an acceptable discriminant validity.

**TABLE 4:** Interfactor correlation matrix of the SACIE-R scale according to sample type.

Factors/dimensions	F1			F2			F3		
	W	S	T	W	S	T	T	S	S
Factor 1- attitudes	*0*.*71*	*0*.*70*	*0*.*72*						
Factor 2- sentiments	−0.14**	–0.04	−0.31**	*0*.*67*	*0*.*67*	*0*.*65*			
Factor 3- concerns	–0.05	–0.04	−0.11*	0.39**	0.38**	0.35**	*0*.*67*	*0*.*69*	*0*.*60*

W = Whole sample; S = Students; T = Teachers; *the correlations are significant (*p* ≤ 05); **(*p* ≤ 0.01). In the diagonal, the values of the AVE square root for each factor are shown, written in italics.

### Attitudes, Sentiments, and Concerns About Inclusive Education of Teachers and Teaching Students

The analysis of the descriptives, the mean differences, and the correlations were carried out with the final model obtained from the previous AFCs.

Based on the results obtained (Table 5), it appeared that both in-service teachers and teacher training students had positive Attitudes toward inclusive education. Specifically, high scores were observed in the attitudes dimension of the SACIE-R (whole sample: *M* = 4.02; *SD* = 0.82; teacher training students; *M* = 3.95; *SD* = 0.89; and in-service teachers *M* = 4.09; *SD* = 0.73), while they present medium-low levels of Sentiments (whole sample: *M* = 2.81; *SD* = 0.98; teacher training students; *M* = 2.69; *SD* = 1.06; and in-service teachers *M* = 2.94; *SD* = 0.88), and Concerns (whole sample: *M* = 2.67*; SD* = 0.89; teacher training students; *M* = 2.42; *SD* = 0.83; and in-service teachers *M* = 2.92; *SD* = 0.88).

**TABLE 5:** Descriptive results of SACIE-R dimensions according to sample type.

	*M*			*SD*		
	W	S	T	W	S	T
*Scale of attitudes, sentiments*						
*and concerns (SACIE-R)*						
Factor 1- *attitudes*	4.02	3.95	4.09	0.82	0.89	0.73
Factor 2- *sentiments*	2.81	2.69	2.94	0.98	1.06	0.88
Factor 3- *concerns*	2.67	2.42	2.92	0.89	0.83	0.88

*M = Mean; SD = Standard Deviation; W = Whole sample; S = Students; T = Teachers.*

Comparing in-service teacher and teacher training students, the highest scores for all three dimensions were found in the in-service teacher sample in Attitudes [*t*(360) = −2.18; *p* = 0.03], although the effect size was small (*d* = 0.17); Concerns [*t*(636) = −3.36; *p* ≤ 0.001], where the effect size was (*d* = 0.15); and Sentiments [*t*(630) = −2.36; *p* = 0.02], where the effect size was also small (*d* = 0.19).

Regarding gender, statistically significant differences (*p* ≤ 0.05) were found for the Attitudes dimension in the student sample but not in the others [*t*(298) = −2.63; *p* ≤ 0.01], although the effect size was small (*d* = 0.15). In general, women seemed to show more positive Attitudes toward IE than men (*M*_*women*_=4.02, *SD*_*women*_=0.88, *M*_*men*_ = 3.70, *SD*_*men*_=0.91).

Regarding differences according to contact during schooling with peers with SENs or having friends or family with some type of disability, no statistically significant differences were found (*p* > 0.05). Nevertheless, slightly higher scores were observed in Attitudes and Concerns for those who did have peers with SEN or those with friends or family with some type of disability.

Likewise, considering the relationship of age with attitudes toward inclusion, a low but significant positive correlation was observed (*p* ≤ 0.01) with the dimensions of the SACIE-R (Attitudes: *r* = 0.11; Sentiments *r* = 0.12; Concerns *r* = 0.22). On the other hand, when considering experience or training related to SEN, low significant correlations were observed (*p* ≤ 0.01) between the frequency of receiving training during the career on attention to diversity, frequency of updating training on attention to diversity, and frequency of teaching to students with disabilities with the dimensions of the SACIE-R. In all cases there was a positive relationship with the Attitudes dimension and negative relationships with Sentiments and Concerns, apart from the frequency with which they had taught students with disabilities and in the Concerns dimension whose correlation was not significant. When considering each sample separately, similar correlations were observed, with slightly higher levels in the case of teachers than students and with the exception of the correlations of the dimensions of the SACIE-R, with respect to the frequency of receiving training on attention to diversity during the career/studies; here, low values were also observed, but they were slightly higher in the case of the students (Table 6).

**TABLE 6:** Matrix of correlations according to subsample (in-service teachers and university students between the dimensions of the SACIE-R and age, variable frequency of training on attention to diversity and teaching pupils with disabilities).

	1			2			3			4			5			6			7			8		
	W	S	T	W	S	T	W	S	T	W	S	T	W	S	T	W	S	T	W	S	T	W	S	T
1	1	1	1																					
2	−0.32**	0.08	−0.31**	1	1	1																		
3	0.32**	0.08	0.06	0.23**	0.60**	0.34**	1	1	1															
4	0.10*	0.05	0.05	0.42**	0.28**	0.42**	0.46**	0.39**	0.53**	1	1	1												
5	0.11**	0.12*	0.05	0.13**	0.18**	0.13*	0.21**	0.24**	0.16**	0.19**	0.09	0.22**	1	1	1									
6	0.12**	–0.01	0.07	−0.18**	−0.17**	−0.13*	−0.16**	−0.15*	−0.19**	−0.11*	−0.14*	−0.17**	−0.17**	−0.14**	–0.04	1	1	1						
7	0.22**	0.07	–0.02	−0.22**	−0.16**	−0.17**	−0.21**	−0.16**	−0.30**	–0.05	–0.11	−0.20**	−0.20**	–0.05	–0.04	0.39**	0.39**	0.35**	1	1	1			
8	0.86**	–	0.86**	−0.29**	–	−0.29**	0.01	–	0.01	0.05	–	0.05	0.01	–	0.01	0.08	–	0.08	−0.11*	–	−0.11*	1	–	1

1 = Age; 2 = Frequency of training on attention to diversity; 3 = Frequency with which you update your training on attention to diversity; 4 = Frequency of updating your training on attention to diversity; 5 = Attitudes; 6 = Sentiments; 7 = Concerns; 8 = Teaching experience; W = whole sample; S = Students; T = Teachers; - = Correlation has not been carried out; **Correlations are statistically significant (*p* ≤ 0.01); *p ≤ 0.05.

## Discussion

Inclusive education is a challenge in the education system, and, although there have been many attempts to incorporate changes, work still needs to be done. Since school inclusion is a key aspect in modern societies, such inclusion seems to be related to greater academic and professional (Messiou et al., 2016) success, a better school environment (Booth and Simón, 2015), and higher levels of well-being (Tardi, 2012). The contributions of this study are mainly threefold: to adapt the SACIE-R scale, which is widely used internationally, to the Spanish context, to analyze the psychometric properties in two subsamples (active teachers and teacher training students), and to analyze the influence of other variables (gender, training, or the level of contact with inclusive education) on attitudes, sentiments and concerns toward inclusive education.

Given the importance of attitudes in predicting inclusion in the classroom, the need has arisen for tools to assess them. To this end, various measurement instruments have been adapted for the Spanish context (González-Gil et al., 2013; Vélez, 2013; Ruiz and Palomino, 2015), but they have focused on more on necessities of inclusion than on assessment to attitudes, sentiments and concerns of teachers about IE. That is why our study has sought to adapt a tool that assesses these dimensions, the SACIE-R, which is widely used worldwide. Among the different instruments available, the SACIE-R (Forlin et al., 2011) is the one with the greatest academic support. The instrument has been widely used with adequate psychometric properties in other countries (Santos and César, 2010; Cansiz and Cansiz, 2018; Murdaca et al., 2018); however, it has never been adapted or validated for the Spanish context nor has it been considered simultaneously for in-service teachers and future teachers (students).

To achieve adequate psychometric properties in both samples in the Spanish context, it was necessary to remove five items from the original scale (Concerns: items 8 and 10, Sentiments: items 4, 13, and 14) sample. The reliability of the Cronbach’s alpha and the CRC (composed reliability) of the three dimensions were adequate. Of the three dimensions of the SACIE-R scale (Attitudes, Sentiments, and Concerns), the factor Attitudes toward IE had the best reliability indices compared to the other dimensions in all the samples, as indicated by previous studies (Santos and César, 2010; Cansiz and Cansiz, 2018; Murdaca et al., 2018). These results were like the original scale (Forlin et al., 2011). The results on validity show good adjustment indices for factorial structure (Forlin et al., 2011) and appeared to justify the internal validity of the instrument in the different samples. The instrument showed better psychometric properties for the whole sample and students sample than the in-service teachers sample.

With respect to the second aim, on the one hand, as expected and as the literature suggested (Lambe et al., 2013), both in-service teachers and teacher-training students had positive attitudes toward IE. Due to the lack of literature comparing the attitudes of students and in-service teachers, it has been considered relevant in this study to analyze the differences between both groups. The results indicated high scores in the attitudes toward IE dimension, and the group of teachers showed the highest scores in all dimensions. This result appears contrary to that depicted in the literature, but it is expected that younger teachers or students have a greater predisposition toward inclusion (Lambe et al., 2013; Tsakiridou and Polyzopoulou, 2014). However, the role of this variable in IE is doubtful, so it will be necessary to carry out more studies that compare different academic levels.

On the other hand, the effects of gender on the differences in SACIE-R were analyzed. Generally, the gender-specific differences were statistically significant for students only in the attitudes dimension, which exhibited higher levels in women. These results appear to be in line with previous studies in the literature (de Boer et al., 2011; Tsakiridou and Polyzopoulou, 2014; Novo-Corti et al., 2015; Efilti and Arslan, 2017).

Thirdly, regarding differences according to contact during schooling with peers with SENs or having friends or family with some type of disability, no statistically significant differences were found. These results do not seem to be in line with the literature (Novo-Corti et al., 2015; Mellado et al., 2017). This is probably due to the way contact is assessed as it has been determined by presenting a binary choice—to have contact or not—instead of quantifying the quantity and quality of that contact. In this sense, the quality of the contact is basic, since superficial contact does not allow deep relationships to be established. Therefore, positive attitudes increase (Mellado et al., 2017) to the extent that both quality and quantity are accounted for when determining contact (Crowson and Brandes, 2014; Gonzalez et al., 2015). Aside from this, when we consider the relationship between age and attitudes toward inclusion, a low but significant positive correlation was observed with the dimensions of the SACIE-R. This relationship could be due to the fact that an increased age is usually related to greater experience and, therefore, higher probabilities of having interacted with students with disabilities and of having trained more in this respect as previous studies have indicated (Forlin et al., 2011; Ghiyasvandian et al., 2014; Loreman et al., 2014; Tsakiridou and Polyzopoulou, 2014; Schmidt and Vrhovnik, 2015).

Finally, when considering experience or training related with SEN, low significant correlations were observed between the frequency of receiving training during the career on attention to diversity, frequency of updating training on attention to diversity, and frequency of teaching to students with disabilities with the dimensions of the SACIE-R; in all cases there was a positive relationship with the Attitudes dimension and negative relations with Sentiments and Concerns. These results make sense if we look at the way the questions are asked in each of these dimensions. The Sentiments and Concerns dimensions evaluate attitudes toward inclusion in a negative sense with higher levels in these dimensions indicating higher levels of Concern (negative thoughts) and nervousness when dealing with individuals from this group. All this seems to be in line with existing literature (Forlin et al., 2011; Loreman et al., 2014; Tsakiridou and Polyzopoulou, 2014), which suggests that both training, education, and experience positively affect attitudes toward educational inclusion.

It is necessary to emphasize the novel dimension of this study because it is the first study of the psychometric properties of the SACIE-R in the Spanish context to simultaneously consider both groups, teachers, and teachers in training. The SACIE-R can be considered a useful, practical tool to evaluate attitudes toward education in different cultures and with different languages, both for teachers in training and teachers alike. Likewise, this is the first study to analyze attitudes toward the inclusion of both groups while considering the effect of age, gender, contact, experience, or training and education by comparing teachers and teachers in training in the Spanish context.

The present study represents considerable progress in offering psychometric evidence that justifies the use of an instrument to measure attitudes toward teacher inclusion in the Spanish context, the SACIE-R, while offering guidelines that should be considered for the design of public and private policies that result in improved teacher training, which in turn improve inclusion in schools. In day-to-day practice, it would be interesting to include in the academic curriculum more inter-disciplinary training on inclusive education in the classroom (Galović et al., 2014; Schmidt and Vrhovnik, 2015), not only from a theoretical point of view but also from a practical perspective. Thus, promoting activities that encourage inclusion in the classroom, such as, for example, the puzzle technique or cooperative tasks (Tsakiridou and Polyzopoulou, 2014; Gale et al., 2017) would aid to reduce prejudice and improve positive attitudes within the school context.

In addition, goals would be to carry out continuous training courses that allow in-service teachers to recycle their knowledge and update their skills (de Boer et al., 2011; Loreman et al., 2014; Irby and Clough, 2015), to offer seminars or meetings between teachers, and to create an environment where they can share their knowledge, doubts, and concerns about IE. Despite the interest in the results obtained, the study is not without limitations. First, it would be interesting to extend this research to other populations worldwide and study the temporal stability of the data in the current population from a longitudinal perspective. Another limitation concerns the type of sampling, of convenience, which does not make it possible to ensure the generalizability of the results obtained. Future research should use probabilistic samples, such as stratified or multi-stratified samples, to ensure better generalizability of the results obtained when considering different types of centers (public or private), their geographical layout, or their size, to cite just a few examples. It could also be of interest to use other external measures of IE as heterocompletion questionnaires or to assess other variables, such as organizational factors (teacher role or supports available in the classroom). In addition, it would have been beneficial to have other types of measures to determine contact that accounted for its quality and quantity. All limitations will be considered in futures studies.

## Conclusion

Prior teacher training and updated training become vital to the better preparation of teachers to work with children with educational needs, increasing their self-confidence and helping them to develop a more positive attitude toward inclusive practices. As for future teachers or teacher training students—it is important to examine their knowledge in relation to the educational context in order to better understand how to produce teachers oriented toward social justice and critical pedagogy. The quality of IE requires that initial teacher training be increasingly linked to social justice and further removed from the dominant, medical, or deficit models.

Therefore, at a practical level, it is important to carry out measures that favor inclusion within the educational sphere. To this end, knowing through this type of tool what the general attitudes of teachers are toward inclusive education can help when developing educational programs or other public or private policies. In this way, the aim is to promote, help, and implement measures that favor students with SENs.

## Data Availability Statement

The datasets generated for this study are available on request to the corresponding author.

## Ethics Statement

The study was approved by the bioethics committee of the Catholic University of Valencia (PRUCV/2015/660). All participants received detailed information about the aims and procedures and were informed of confidentiality.

## Author Contributions

All authors listed have made a substantial, direct and intellectual contribution to the work, and approved it for publication.

## Conflict of Interest

The authors declare that the research was conducted in the absence of any commercial or financial relationships that could be construed as a potential conflict of interest.
